# Subacute Haemorrhagic Pituitary Apoplexy Leading to Secondary Adrenal Insufficiency: A Case Report

**DOI:** 10.7759/cureus.106333

**Published:** 2026-04-02

**Authors:** Rashaan Jackson-Wade, Orlaith Fogarty, Nyein Nge Nge, Taofeek Ojewuyi

**Affiliations:** 1 Internal Medicine, Southend University Hospital, Southend-on-Sea, GBR; 2 Acute Medicine/General Medicine, Southend University Hospital, Southend-on-Sea, GBR; 3 Endocrinology, Diabetes, and Metabolism, Southend University Hospital NHS Foundation Trust, Southend-on-Sea, GBR; 4 Diabetes and Endocrinology, Mid and South Essex NHS Foundation Trust, Southend-on-Sea, GBR

**Keywords:** anterior pituitary hormone, anterior pituitary hypofunction, brain tumor, central adrenal insufficiency, hba1c, pituitary adenoma, pituitary hemorrhage

## Abstract

Pituitary apoplexy is an acute and potentially life-threatening complication of pituitary adenomas caused by haemorrhage or infarction within the gland. Clinical presentation may be non-specific, creating diagnostic difficulty and increasing the risk of delayed recognition, particularly when symptoms evolve subacutely.

We report a case of a 52-year-old postmenopausal female with type 2 diabetes who presented with a six-week history of progressive fatigue, weakness, headache, nausea, weight loss, and unexpectedly improved glycaemic control. Initial community blood testing demonstrated an undetectable serum cortisol level, prompting urgent hospital admission. Further evaluation revealed panhypopituitarism, and magnetic resonance imaging demonstrated a haemorrhagic pituitary macroadenoma with suprasellar extension and optic chiasmal compression.

The patient was treated promptly with intravenous hydrocortisone, followed by oral glucocorticoid replacement and levothyroxine, resulting in rapid clinical improvement. This case highlights the diagnostic challenge of pituitary apoplexy presenting with subacute constitutional symptoms and underscores the importance of early recognition and timely multidisciplinary management to prevent life-threatening complications.

## Introduction

Pituitary macroadenomas are relatively common intracranial tumours that account for approximately 10-15% of all intracranial tumours and are typically benign, non-functioning, and indolent [1]. A serious complication of these tumours is pituitary apoplexy (PA), a rare but potentially life-threatening endocrine emergency [2].

PA occurs in approximately 2-10% of pituitary adenomas, with an estimated annual incidence of one per 1,000,000 [1,2]. It results from acute haemorrhage or infarction of the pituitary gland, most commonly in patients with a pre-existing macroadenoma [3].

Endocrine dysfunction is common in PA. Up to 80% of patients develop a deficiency of at least one anterior pituitary hormone [1]. Growth hormone deficiency is the most frequent, followed by adrenocorticotropic hormone (ACTH) deficiency, which occurs in approximately 70% of cases [1]. ACTH deficiency is the most clinically urgent abnormality and may lead to life-threatening acute adrenal insufficiency [4].

Initial management focuses on haemodynamic stabilisation, correction of fluid and electrolyte disturbances, and urgent glucocorticoid replacement [2,5]. The optimal management of the underlying adenoma remains debated. Surgical intervention is recommended for patients with progressive neurological or visual deterioration, whereas conservative management may be appropriate in clinically stable patients [4-7].

This report describes a 52-year-old female presenting with lethargy, weakness, headache, and weight loss who was subsequently found to have a haemorrhagic pituitary macroadenoma.

## Case presentation

A 52-year-old postmenopausal female with type 2 diabetes and a previous cholecystectomy presented to her general practitioner with a six-week history of progressive generalised fatigue, weakness, and daily headaches. Her symptoms worsened significantly over the week, causing a functional decline and leaving her unable to attend work. The headaches were sharp, non-radiating, without visual disturbance, and partially responsive to paracetamol.

She additionally reported nausea, reduced appetite, and unintentional weight loss of 5 kg over six weeks. Notably, she observed marked improvement in glycaemic control despite no changes to her diet or medications. Her regular medications included gliclazide, metformin, empagliflozin, and pravastatin. She was two years postmenopausal and not receiving hormone replacement therapy.

Initial blood tests were done in the morning before admission, as seen in Table 1. Tests demonstrated a cortisol level of <11 nmol/l (185-624), thyroid-stimulating hormone (TSH) level of 3.17 mU/L (0.3-5.0), and free T4 level of 4.4 pmol/L (7.9-16.0). Also of note, her glycosylated haemoglobin (HbA1c) had improved to 49 mmol/mol (20-42) from 67 mmol/mol. In view of this result, she was advised to attend the emergency department for urgent assessment.

**Table 1:** Anterior pituitary hormone profile on admission. FSH: follicle-stimulating hormone; IGF-1: insulin-like growth factor 1; LH: luteinizing hormone; GH: growth hormone; ACTH: adrenocorticotropic hormone; TSH: thyroid-stimulating hormone.

Investigation	Result	Reference range
FSH (U/L)	4.0	16.7-113.6
IGF-1 (nmol/L)	5.7	6.2-27.2
Prolactin (mU/L)	352	58-416
TSH (mU/L)	3.17	0.3-5.0
Free T4 (pmol/L)	4.4	7.9-16.0
LH (IU/L)	Haemolysed	-
Oestradiol	<55	-
GH (ug/L)	No result available	-
ACTH (ng/L)	No result available	-

An ACTH sample was obtained before administration of hydrocortisone; however, due to a sampling error, the result was unfortunately unavailable. In this acute setting, immediate glucocorticoid replacement was prioritised over comprehensive endocrine testing due to the clinical suspicion of adrenal insufficiency. Dynamic testing, such as an insulin tolerance test or metyrapone test, was considered but deferred during the acute phase and planned for outpatient endocrine follow-up once the patient was clinically stable.

Subsequent hormonal evaluation demonstrated deficiencies in multiple anterior pituitary hormones, supporting the diagnosis of panhypopituitarism without the need for dynamic testing. She was haemodynamically stable. Her observations were normal, and she was normotensive. Her visual field assessment via confrontation was normal; neurological examination showed symmetrical proximal muscle weakness without other deficits. The rest of her examination was unremarkable.

Investigation

**Figure 1:**
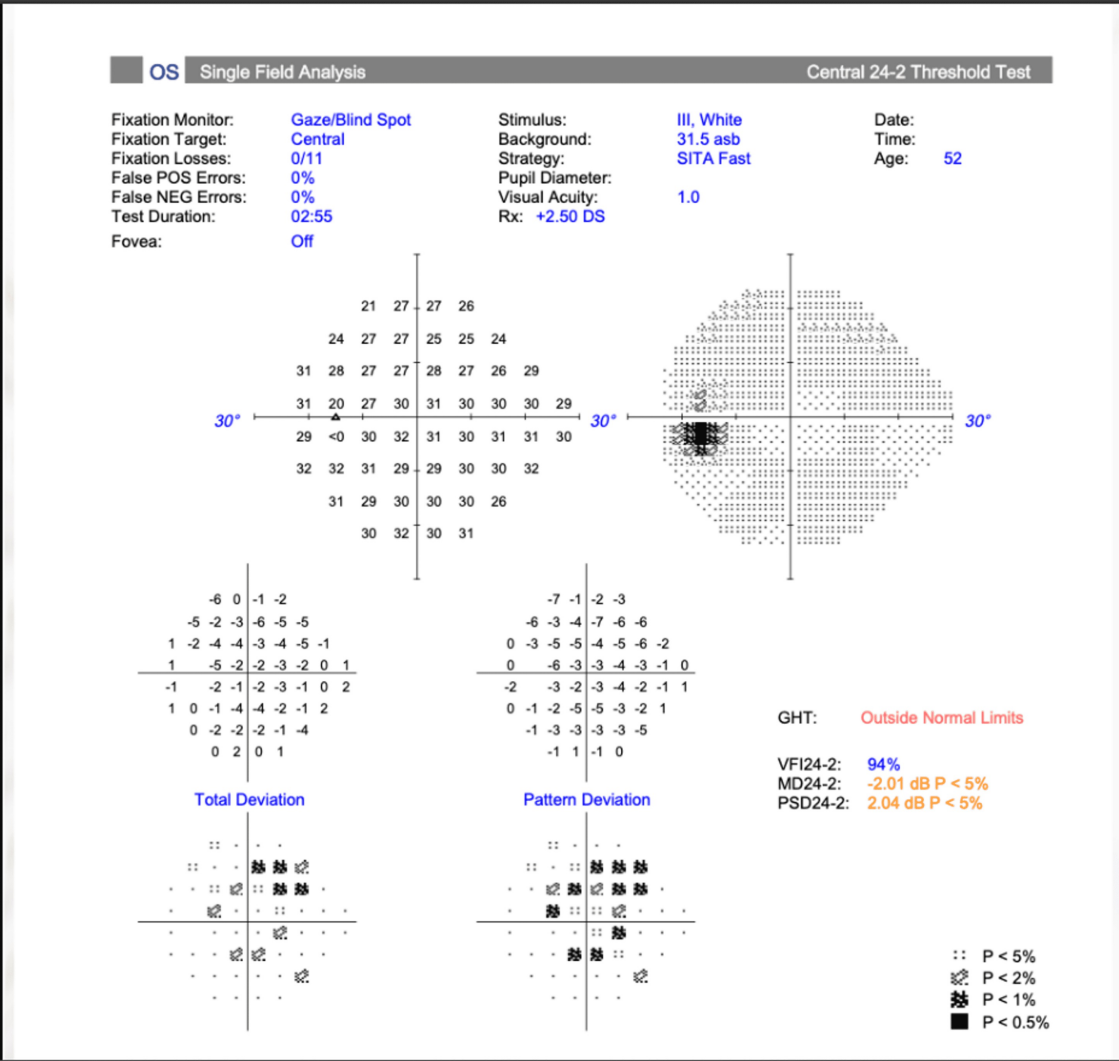
Left visual field test demonstrating full and normal visual field.

**Figure 2:**
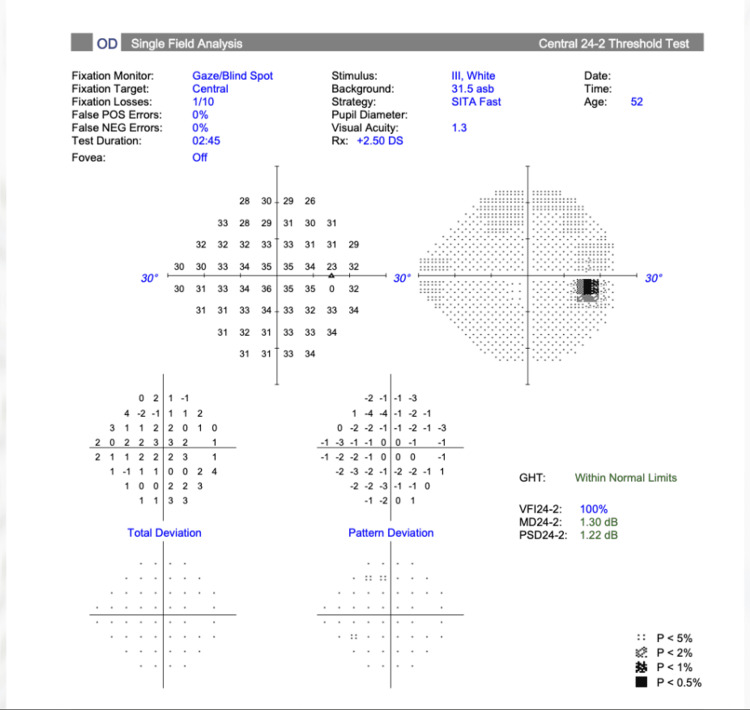
Right visual field test demonstrating full and normal visual field.

Initial anterior pituitary hormone profile (Table 1) indicated panhypopituitarism. Formal Humphrey visual field testing demonstrated full and normal visual fields in both eyes (Figures 1, 2).

**Figure 3:**
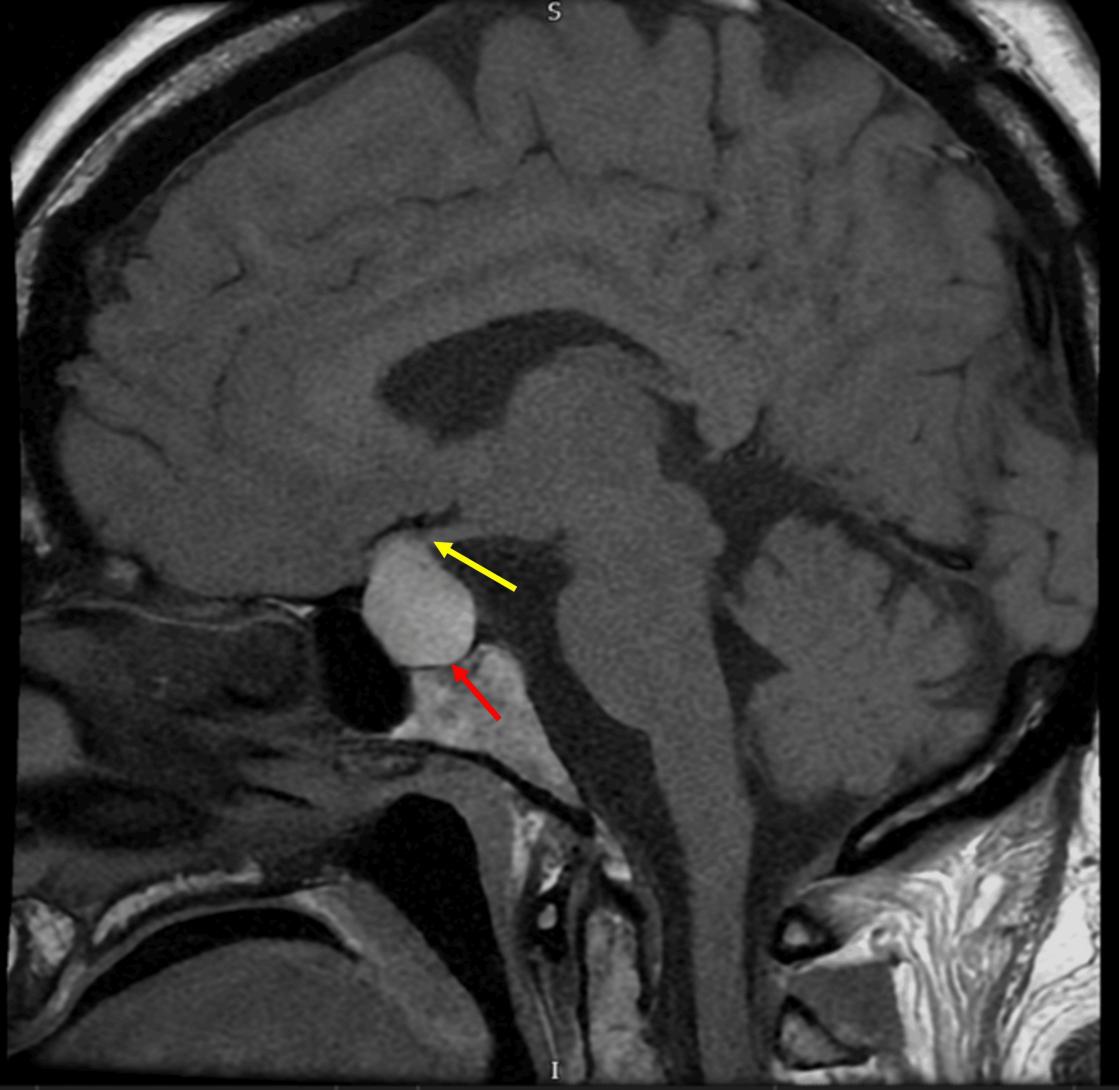
Contrast‑enhanced T1‑weighted sagittal MRI demonstrated a 21 × 13 mm sellar mass (red arrow) with high T1 signal intensity and no measurable contrast enhancement, showing suprasellar extension and compression of the optic chiasm (yellow arrow).

**Figure 4:**
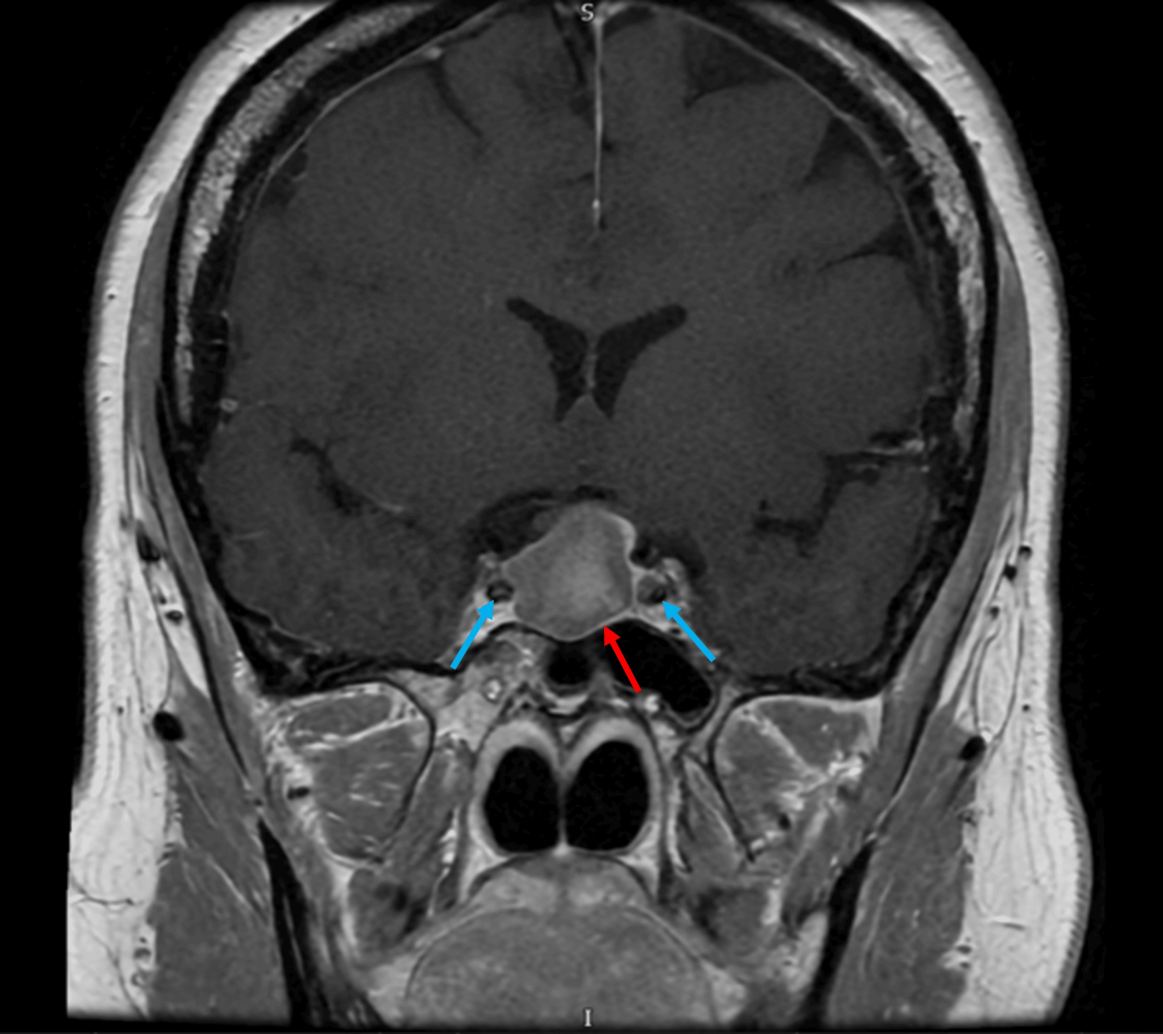
T1‑weighted, contrast‑enhanced coronal MRI demonstrated a 21 × 13 mm sellar mass (red arrow) with high T1 signal intensity, no measurable contrast enhancement, and suprasellar extension abutting both internal carotid arteries (blue arrows).

CT of the head demonstrated a suprasellar mass measuring 24 × 22 × 14 mm with mostly uniform attenuation. No acute intracranial pathology was identified. MRI of the pituitary with contrast demonstrated a sellar mass measuring 21 × 13 mm with suprasellar extension, displacing the optic chiasm and abutting both internal carotid arteries. The lesion demonstrated homogeneous T1 hyperintensity without contrast enhancement, indicating intratumoural haemorrhage. Figure 3 shows the sagittal view demonstrating chiasmal compression. Figure 4 shows the coronal view demonstrating the abutment of the internal carotid arteries.

Treatment

She was started on stress‑dose hydrocortisone with a 100 mg intravenous bolus, followed by another 100 mg the next morning (totalling 200 mg in 24 hours), then transitioned to oral hydrocortisone at 20 mg on waking, 10 mg at noon and 10 mg at 17:00. Levothyroxine 50 µg daily was added once glucocorticoid replacement was established.

Outcome and follow-up

Within 48 hours of initiating treatment, there was marked clinical improvement with resolution of nausea and headache, significant improvement in energy levels, and restoration of appetite. Throughout her admission, she was monitored for any neuro‑ophthalmic or neurological symptoms, and was asymptomatic regarding these. She was reinitiated on her anti-hyperglycaemic medications before discharge.

Her case was discussed with the neurosurgical team at initial presentation, and she was discussed in the pituitary multidisciplinary team (MDT). As she did not have any neuro‑ophthalmic or neurological symptoms, and she was fully conscious, the MDT decided that she should be managed conservatively, with a plan of follow‑up endocrine and outpatient neurosurgical review.

From an ophthalmology perspective, she was asymptomatic, her formal visual field test was normal and showed no evidence of bitemporal hemianopia, so she was discharged from the ophthalmology clinic with an open appointment should visual symptoms arise.

Her glucocorticoid regimen at discharge was weaned over three days to 10 mg on waking, 5 mg at noon, and 5 mg in the evening as her maintenance dose.

**Figure 5:**
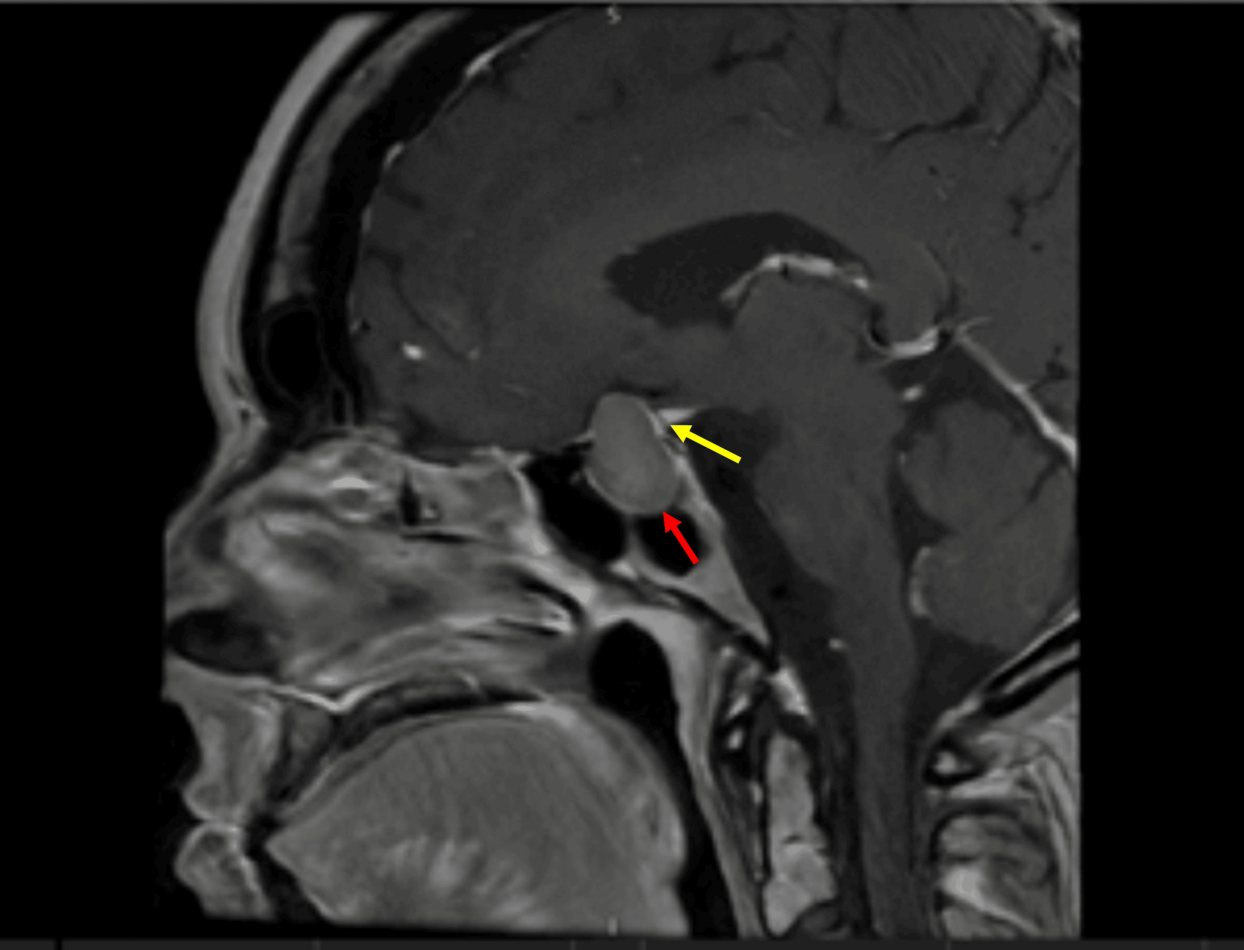
Contrast-enhanced sagittal view of the MRI scan eight months after initial presentation. The size of the tumour (red arrow) was unchanged, and it continued to remain in contact with the optic chiasm (yellow arrow).

**Figure 6:**
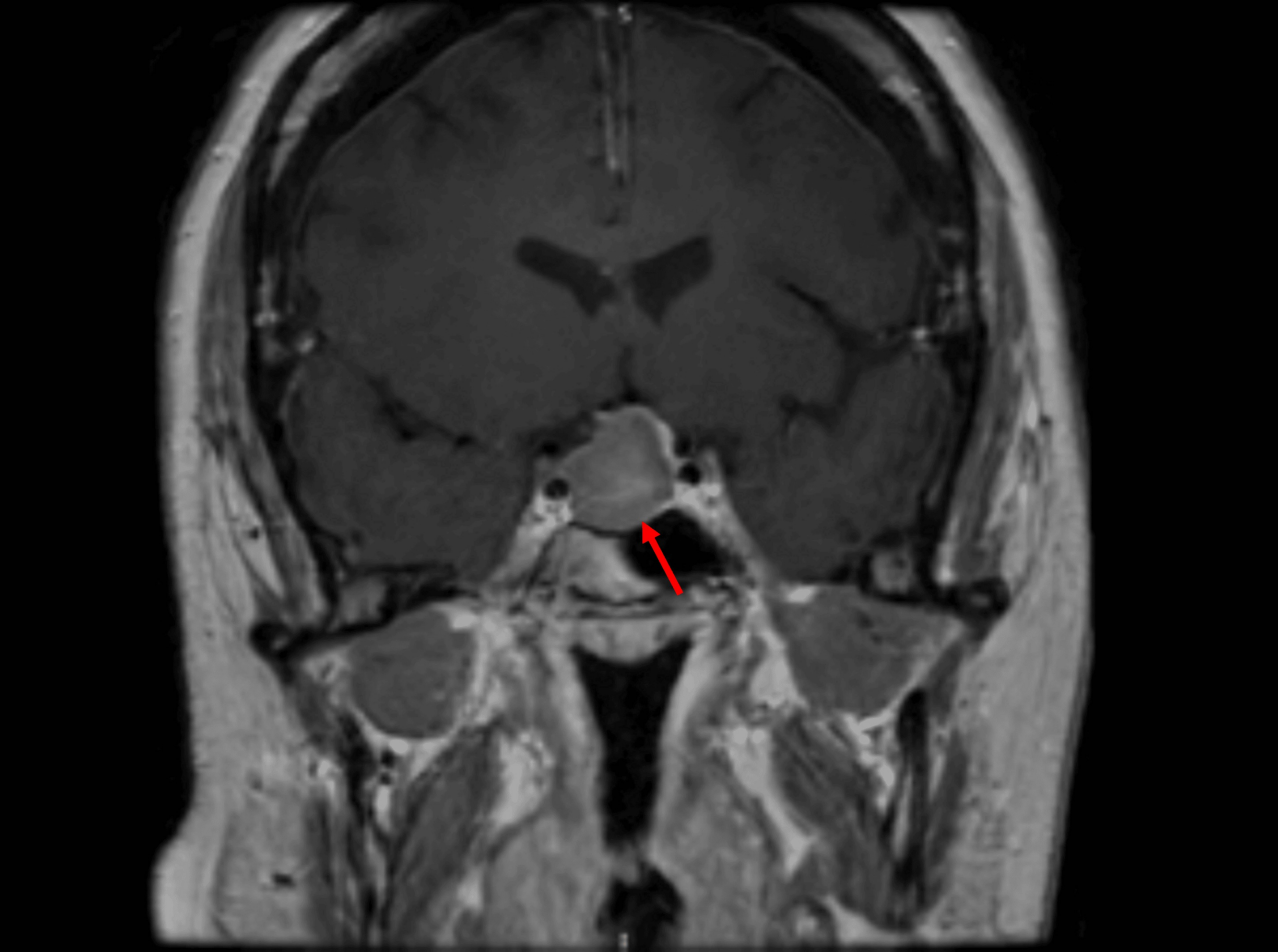
Contrast-enhanced coronal view of the MRI pituitary scan eight months after initial presentation. The appearance of the pituitary gland (red arrow) remained stable, with no change in size or morphology of the mass.

Following discharge, the patient was reviewed in the neurosurgical clinic 20 weeks later. The review concluded there was still no recommendation for immediate surgical intervention, but the patient required an MRI to assess for interval changes. Follow-up imaging eight months later demonstrated no interval change in tumour size or morphology. Figure 5 shows the sagittal view, and Figure 6 shows the coronal view. She is waiting for a further neurosurgery follow-up appointment following her repeat MRI.

At her six‑month endocrinology review, she remained symptom‑free with no visual field complaints. Repeat testing demonstrated ongoing deficiencies across multiple anterior pituitary hormones, as seen in Table 2, reaffirming panhypopituitarism without requiring dynamic stimulation tests.

**Table 2:** Follow-up endocrinology testing at six months demonstrated persistent hypopituitarism. FSH: follicle-stimulating hormone; IGF-1: insulin-like growth factor 1; LH: luteinizing hormone; ACTH: adrenocorticotropic hormone; TSH: thyroid-stimulating hormone.

Investigation	Result	Reference range
ACTH (ng/L)	4	<50
Cortisol (nmol/L)	33	186-624
IGF-1 (nmol/L)	13.4	6.2 - 27.2
FSH (U/L)	5	16.7 - 113.6
LH (IU/L)	1.9	10.9 – 58.6
Oestradiol	<55	
Prolactin (mU/L)	306	58 - 416
TSH (mU/L)	1.55	0.3-5.0
Free T4 (pmol/L)	12.0	7.9-16.0

## Discussion

This 52-year-old post-menopausal female presented with a six-week history of fatigue, weakness, and headache. Biochemical evaluation revealed panhypopituitarism, and MRI demonstrated a haemorrhagic pituitary macroadenoma. Of note, her HbA1c improved despite untreated hypothyroidism and no reported changes in medication or diet.

The clinical presentation of PA varies depending on the extent and rapidity of haemorrhage or infarction. Although our patient presented with subacute symptoms developing over six weeks with no neurological or ophthalmological symptoms, most cases present suddenly with severe headache and diplopia or ophthalmoplegia [1,2]. In a 2024 review, headache was present in about 89% of cases and ophthalmoplegia in 78%, as seen in Table 3 [1]. Sudden onset headache alongside neurology symptoms can be associated with other life-threatening conditions, such as subarachnoid haemorrhage, meningitis, and hypertensive encephalopathy, and should also be considered, as seen in Table 4. These conditions can be distinguished from PA by the absence of pituitary or endocrine dysfunction. Other symptoms may include fatigue, weight loss, anorexia, myalgia, abdominal pain, and severe hypotension due to acute cortisol deficiency. These signs reflect the degree of compression of the pituitary gland and adjacent neuro‑ophthalmic structures [1].

**Table 3:** Common presentations of pituitary apoplexy, alongside the frequency of presentation.

Presentation	Frequency [1]	
Sudden severe headache	89%	Typically retro‑orbital or frontal, often the first symptom
Ophthalmoplegia/diplopia	78%	Most commonly due to cranial nerve III palsy, followed by IV, then VI palsies
Vomiting/nausea	69%	Associated with headache
Visual field defects	64%	Often bitemporal hemianopia
Decreased visual acuity	52%	Can progress to blindness
Gonadotropin deficiency	75%	At diagnosis, most patients have gonadotropin deficiency
Adrenocorticotropic hormone deficiency	70%	Often associated with hyponatraemia
Thyrotropin deficiency	60%	-

**Table 4:** Common differentials to pituitary apoplexy, with the key clinical and investigation differences.

Differential diagnosis	Key clinical difference	Key investigation
Subarachnoid haemorrhage	Classical sudden thunderclap headache, with neck stiffness, meningism, and rapid deterioration; no pituitary dysfunction [1,8]	Non‑contrast CT or lumbar puncture shows blood in the subarachnoid space; confirmation with MRI with no sellar mass seen [8]
Venous sinus thrombosis	Headache with cranial‑nerve palsies and fever; often associated with infection or hypercoagulable states; no pituitary dysfunction [1,8]	Magnetic resonance venography shows thrombosis of venous sinuses; MRI/CT cavernous sinus involvement without an adenoma [1]
Internal carotid artery aneurysm	Typically, sudden headache or cranial‑nerve palsies with no pituitary dysfunction [1,8]	MRI shows a round lesion with flow void; a partially thrombosed aneurysm appears hyperintense on T1; magnetic resonance angiography confirms vascular nature.
Rathke cleft cyst	Usually presents with chronic headaches or endocrine symptoms rather than acute [8]	MRI shows midline cyst with proteinaceous contents, giving T1 hyperintensity; characteristic intracystic hypointense nodule on T2; no blood–fluid level [8]
Encephalitis/meningitis	Fever, neck stiffness, altered mental status, and diffuse neurological signs; lacks endocrine symptoms [8]	CSF analysis shows elevated neutrophils and positive cultures; MRI can show meningeal enhancement; no pituitary mass [8]
Hypertensive encephalopathy	Headache, confusion, seizures and visual disturbances in the context of severe hypertension; not related to pituitary dysfunction [1,8]	MRI shows posterior vasogenic oedema without a sellar lesion [8]

PA secondary to tumour infarction generally presents more insidiously and with milder symptoms than haemorrhagic PA, although long-term hormonal outcomes are similar [6]. While haemorrhage is often confined to the tumour, extension into the subarachnoid space may produce meningeal irritation. Large macroadenomas with suprasellar extension can rarely result in obstructive hydrocephalus [4].

The most serious acute complication is secondary adrenal insufficiency, which may present as adrenal crisis characterised by hypotension, hypoglycaemia, hyponatraemia, and hyperkalaemia [1,2,6]. Precipitating factors are identified in approximately 30-40% of cases [2,3]. These include hypertension, diabetes mellitus, large non-functioning adenomas (particularly silent corticotroph adenomas), and aneurysm rupture [2,3,7]. Patients with known pituitary tumours should therefore be counselled regarding symptoms of PA to facilitate early recognition [2,3,7].

Non-contrast CT is often the initial imaging modality in the acute setting due to availability, and may demonstrate a hyperdense sellar mass suggestive of haemorrhage [6,8]. However, its sensitivity for detecting infarction or tumour necrosis is limited, and as represented in our case, a non-contrast CT cannot be used to rule out haemorrhage and apoplexy [6,8]. MRI is the preferred diagnostic modality, with a sensitivity of approximately 90%, as it better delineates haemorrhage, necrosis, oedema, and optic chiasmal compression [5].

Empirical glucocorticoid therapy should be initiated immediately in all suspected cases, even in the absence of overt adrenal crisis [1,9]. Recommended treatment consists of an intravenous hydrocortisone bolus of 100-200 mg, followed by 50-100 mg every six hours or continuous infusion at 2-4 mg/hour [1,9]. Thyroid hormone replacement must only be commenced after adequate glucocorticoid replacement to avoid precipitating adrenal decompensation. This is critical as thyroxine increases cortisol metabolism and basal metabolic rate. Thus, initiating thyroxine before glucocorticoids will reduce the already limited circulating endogenous cortisol whilst also increasing the requirement for glucocorticoids, ultimately worsening adrenal insufficiency and putting patients at risk of adrenal crisis [9]. Additionally, high-dose glucocorticoids also play a role in reducing inflammation and oedema of the pituitary and surrounding structures [1].

Management of PA requires coordinated multidisciplinary input from endocrinology, neurosurgery, neuroradiology, and neuro-ophthalmology [1,5,6]. The choice between conservative management alone vs. surgery for PA is strongly contested, particularly for patients with mild symptoms and no progressive deficits [1,5,9]. The contention lies as to whether and when surgery is indicated. Partly, this contention is due to the rarity of the condition, which makes it difficult to generate randomised controlled trials.

A recent review found that 30% of reported PA cases were managed conservatively [1]. Conservative management consists of hormone replacement, haemodynamic stabilisation, and close neurological and ophthalmological monitoring, with a low threshold for repeat MRI if symptoms worsen. Current guidelines recommend this approach initially for all patients and as sole therapy in subacute cases or in patients with mild or no neurological deficits [1,9].

Under current guidelines, urgent surgery is recommended in patients with progressive neurological or visual deficits, although the overall superiority of surgical versus conservative management remains uncertain for stable patients [1,9]. Reservations regarding decompression stem from the risks of transsphenoidal surgery, including CSF leak, permanent diabetes insipidus, and loss of normal pituitary tissue. This risk has not been shown to have superior visual or endocrine outcomes for early surgery [9]. Regarding recovery, a recent review has shown no significant difference between the two management options for visual fields and hormonal recovery, as seen in Table 5 [1].

**Table 5:** Recovery rates for visual field and endocrine function in surgical versus conservative management of pituitary apoplexy. Source: Iglesias [1].

Outcome	Surgical management recovery rate	Conservative management recovery rate
Visual field recovery	76%	79%
Endocrine function recovery	23%	23%

Regarding our patient’s diabetes, her HbA1c improved despite untreated hypothyroidism and no intentional changes to her diabetes management. Cortisol deficiency increases insulin sensitivity and reduces hepatic gluconeogenesis, which lowers blood glucose and increases peripheral glucose uptake [10]. However, such HbA1c improvements in subacute pituitary are rarely described in the literature, and our case highlights a potential diagnostic clue when unexplained glycaemic improvement accompanies fatigue and weight loss.

In this case, given stable visual function and clinical improvement with medical therapy, a conservative multidisciplinary approach was adopted. Surgery would be reconsidered if she developed new or worsening visual or neurological deficits. She underwent daily neurological and visual checks during admission, was discharged with an open ophthalmology appointment and scheduled neurosurgical follow‑up, and remains under endocrine and neurosurgical surveillance.

## Conclusions

Pituitary apoplexy may present subacutely with vague, non-specific symptoms and can be missed in the absence of classical neuro-ophthalmic signs. Clues such as unexplained improvement in glycaemic control in patients with diabetes, accompanied by fatigue, weight loss, nausea, and headache, should raise suspicion for cortisol deficiency and secondary adrenal insufficiency, particularly when other pituitary hormone deficiencies are identified. Early morning serum cortisol serves as an essential screening test, and a markedly low or undetectable level warrants immediate glucocorticoid replacement.

## References

[1] Iglesias P (2024). Pituitary apoplexy: an updated review. J Clin Med.

[2] Briet C, Salenave S, Bonneville JF, Laws ER, Chanson P (2015). Pituitary apoplexy. Endocr Rev.

[3] Biousse V, Newman NJ, Oyesiku NM (2001). Precipitating factors in pituitary apoplexy. J Neurol Neurosurg Psychiatry.

[4] Zayour DH, Selman WR, Arafah BM (2004). Extreme elevation of intrasellar pressure in patients with pituitary tumor apoplexy: relation to pituitary function. J Clin Endocrinol Metab.

[5] Goyal P, Utz M, Gupta N, Kumar Y, Mangla M, Gupta S, Mangla R (2018). Clinical and imaging features of pituitary apoplexy and role of imaging in differentiation of clinical mimics. Quant Imaging Med Surg.

[6] Dubuisson AS, Beckers A, Stevenaert A (2007). Classical pituitary tumour apoplexy: clinical features, management and outcomes in a series of 24 patients. Clin Neurol Neurosurg.

[7] Fu J, Li Y, Wu L (2021). Pituitary hemorrhage in pituitary adenomas treated with gamma knife radiosurgery: incidence, risk factors and prognosis. J Cancer.

[8] Boellis A, di Napoli A, Romano A, Bozzao A (2014). Pituitary apoplexy: an update on clinical and imaging features. Insights Imaging.

[9] Rajasekaran S, Vanderpump M, Baldeweg S (2011). UK guidelines for the management of pituitary apoplexy. Clin Endocrinol (Oxf).

[10] Lee SC, Baranowski ES, Sakremath R, Saraff V, Mohamed Z (2023). Hypoglycaemia in adrenal insufficiency. Front Endocrinol (Lausanne).

